# Long COVID awareness and receipt of medical care: a survey among populations at risk for disparities

**DOI:** 10.3389/fpubh.2024.1360341

**Published:** 2024-05-30

**Authors:** Kimberly A. Fisher, Kathleen M. Mazor, Mara M. Epstein, Lydia Goldthwait, Hiba Abu Ghazaleh, Yanhua Zhou, Sybil Crawford, Jai Marathe, Benjamin P. Linas

**Affiliations:** ^1^Division of Health Systems Science, Department of Medicine, UMass Chan Medical School, Worcester, MA, United States; ^2^Division of Pulmonary and Critical Care Medicine, Department of Medicine, UMass Chan Medical School, Worcester, MA, United States; ^3^Tan Chingfen Graduate School of Nursing, UMass Chan Medical School, Worcester, MA, United States; ^4^Boston Medical Center, Boston, MA, United States; ^5^Section of Infectious Diseases, Department of Medicine, Boston University School of Medicine, Boston, MA, United States; ^6^Boston University School of Public Health, Boston, MA, United States

**Keywords:** COVID-19, long COVID, health disparities, awareness, national survey

## Abstract

**Introduction:**

The COVID-19 pandemic has been characterized by disparities in disease burden and medical care provision. Whether these disparities extend to long COVID awareness and receipt of medical care is unknown. We aimed to characterize awareness of long COVID and receipt of medical care for long COVID symptoms among populations who experience disparities in the United States (US).

**Methods:**

We conducted a cross-sectional survey among a national sample of US adults between January 26–February 5, 2023. We surveyed approximately 2,800 adults drawn from the Ipsos probability-based KnowledgePanel® who identify as White, Black, or Hispanic, with over-sampling of Black, Hispanic, and Spanish-proficient adults. Awareness of long COVID was assessed with the question, “Have you heard of long COVID? This is also referred to as post-COVID, Long-haul COVID, Post-acute COVID-19, or Chronic COVID.” Respondents reporting COVID-19 symptoms lasting longer than 1 month were classified as having long COVID and asked about receipt of medical care.

**Results:**

Of the 2,828 respondents, the mean age was 50.4 years, 52.8% were female, 40.2% identified as Hispanic, 29.8% as Black, and 26.7% as White. 18% completed the survey in Spanish. Overall, 62.5% had heard of long COVID. On multivariate analysis, long COVID awareness was lower among respondents who identified as Black (OR 0.64; 95% CI 0.51, 0.81), Hispanic and completed the survey in English (OR 0.59; 95% CI 0.46, 0.76), and Hispanic and completed the survey in Spanish (OR 0.31, 95% C.I. 0.23, 0.41), compared to White respondents (overall *p* < 0.001). Long COVID awareness was also associated with educational attainment, higher income, having health insurance, prior history of COVID-19 infection, and COVID-19 vaccination. Among those reporting symptoms consistent with long COVID (*n* = 272), 26.8% received medical care. Older age, longer symptom duration and greater symptom impact were associated with receipt of medical care for long COVID symptoms. Of those who received care, most (77.8%) rated it as less than excellent on a 5-point scale.

**Discussion:**

This survey reveals limited awareness of long COVID and marked disparities in awareness according to race, ethnicity, and language. Targeted public health campaigns are needed to raise awareness.

## Introduction

During the initial phase of the COVID-19 pandemic, it became clear that some individuals who survived the acute phase of infection with SARS-CoV-2, the virus that causes COVID-19, do not experience a full recovery, but instead continue to experience symptoms weeks and months later. Long COVID, also known as post-acute sequelae of SARS-CoV-2 infection (PASC), is defined by the continuation, exacerbation, or new development of any one of a variety of wide-ranging, multisystem symptoms at least 4 weeks after initial COVID-19 infection and is not dependent on the severity of initial infection (1, 2). Commonly reported symptoms of long COVID include fatigue, respiratory and cardiac symptoms, neurological issues including brain fog, and joint pain (1–4). Lingering debilitating symptoms can impede a patient’s ability to work and negatively impact their quality of life (5, 6). In addition, some patients suffer from symptoms that are difficult to diagnose, resulting in delayed receipt of appropriate care (2). With the estimated prevalence of long COVID ranging from 6.9–40% of all people diagnosed with COVID, long COVID is a major public health concern (7–9).

The COVID-19 pandemic has been characterized by significant racial, ethnic, and socioeconomic disparities in the United States (US), with higher COVID-19 case rates, hospitalizations, and deaths among Black, Latino, and socioeconomically disadvantaged communities (10–15). Limited English proficiency has also emerged as a risk factor for COVID-19 infection, highlighting the limited reach of public health information among non-English speaking communities (16). In addition to disparities in disease burden, the COVID-19 pandemic has laid bare structural disparities in access to medical care, including COVID-19 diagnostics (17), vaccines (18), and treatment (19). The potential for these existing disparities in healthcare access to compound the frequent challenges that have been described among long COVID patients in accessing medical care (20, 21) is concerning. A few qualitative studies have documented the experience of long COVID among members of racial and ethnic minorities (22–24), but disparities in awareness of long COVID and utilization patterns of medical care for long COVID among a broader population have not been examined.

The goal of this study was to characterize awareness of long COVID and receipt of medical care for long COVID among populations at risk of healthcare disparities. We also describe the experience of those patients who received care for long COVID.

## Methods

### Study design

This is an observational, cross-sectional study, using a self-administered online survey.

### Setting

We surveyed a sample of adults residing in the United States via the Ipsos KnowledgePanel®, an online panel representative of the entire US population. Ipsos recruits panel members using probability-based sampling methods; potential panel members are provided with internet access and hardware if needed. Both English and Spanish speakers are recruited to the KnowledgePanel®.

### Participants

For the present survey, we recruited KnowledgePanel® members who identified as White, Black, and/or Hispanic. We oversampled Black, Hispanic, and Spanish-proficient Hispanic adults to enhance racial and ethnic diversity and ensure adequate representation of non-English speaking adults in the sub-sample of respondents with symptoms consistent with long COVID. In addition, we oversampled Spanish-proficient Hispanic adults to confirm the findings of a prior pilot survey that suggested there may be disparities in long COVID awareness among Spanish speakers. Ipsos sent email invitations to panel members, with reminder emails to non-responders three and 7 days after the initial invitation. The survey was available in English and Spanish. Participants received an incentive valued at ~$5 for their participation. We conducted the survey between January 26 and February 5, 2023.

### Variables

The primary outcome was awareness of long COVID, assessed with the question, “Have you heard of long COVID? This is also referred to as post-COVID, Long-haul COVID, Post-acute COVID-19, or Chronic COVID.” A secondary outcome was report of symptoms consistent with long COVID, defined by the presence of COVID-19 symptoms lasting longer than 1 month, among respondents with a history of COVID-19 infection. An additional secondary outcome was receipt of medical care among respondents with symptoms consistent with long COVID.

Co-variates included sociodemographic items (e.g., race, ethnicity, education, insurance status), beliefs related to long COVID, history of COVID-19 infection, COVID-19 vaccination status, and long COVID symptom duration and impact (among respondents with symptoms consistent with long COVID). Respondents who did not receive medical care for symptoms of long COVID were asked why they did not pursue care or were not able to see a provider for their long COVID symptoms (open-ended response). Those who received medical care for their long COVID symptoms were asked to provide an overall rating of the care they received with response options: excellent; very good; good; fair; and poor. Respondents who rated their care as less than excellent were asked the open-ended question, “Please tell us how your care fell short, and what should be done differently in the future.” The full set of items included in the survey for the present study are provided in Supplementary file S1. The survey items were pre-tested in a small (*n* ~ 300) study with a local sample. Demographic information (e.g., age, education level, household income) was provided by members when they joined the panel.

We constructed five categories to reflect participants’ reported race, ethnicity, and language in a single variable: White, non-Hispanic; Black, non-Hispanic; Hispanic, survey completed in English; Hispanic, survey completed in Spanish; other or more than one race, non-Hispanic.

For the question “Have you had COVID-19,” we combined ‘Yes, had it once’ and ‘Yes, had it more than once’ into a single category. We dichotomized insurance status into ‘no insurance’ and ‘any insurance’. To examine care seeking related to long COVID, we categorized participants as having symptoms consistent with long COVID if they responded that they had COVID-19 at least once and they had experienced COVID-19 symptoms that lasted for at least 1 month. We further categorized participants who met these criteria as (a) having sought and received medical care or (b) having not received medical care at the time of the survey (not sought or sought care but not received). We combined the latter two groups (not sought care and sought care but not received) because our goal was to predict receipt of care and describe the experience of care among those who received it. The group who sought care but had not received it was small (*n* = 20).

Spanish language responses to the open-ended items (e.g., Why did not you pursue care for your long COVID symptoms?) were professionally translated into English for analysis.

### Statistical methods

We used means, frequencies, and percentages to summarize participant characteristics. We computed chi-square statistics to examine the associations between participant characteristics and awareness of long COVID. To create a parsimonious model predicting long COVID awareness, we used a backward stepwise approach starting with standard demographic variables (race/ethnicity/language, age, gender, and education), and all additional variables that we hypothesized to be associated with long COVID awareness that also showed some evidence of possible association in the bivariate analyses (i.e., resulted in *p* < 0.20). The final model retained race/ethnicity/language, age, and gender independent of statistical significance. We used a similar approach to identify factors associated with receipt of medical care among respondents who reported symptoms consistent with long COVID. In addition to retaining standard demographics independent of statistical significance, this model also retained insurance status (no insurance vs. any insurance) given its relevance to receipt of care. For participants who received care, we computed descriptive statistics for their perceptions of care (taken seriously and satisfaction), whether they were diagnosed with long COVID and whether they were referred elsewhere for further care. Statistical analyses were performed with SAS version 9.4 (SAS Institute Inc., Cary, NC, United States) and SPSS version 28.0.1.0 (IBM SPSS Statistics, Armonk, NY, United States).

### Qualitative analyses

We used conventional content analysis to separately analyze responses to each open-ended item (25). At least two authors reviewed a subset of the responses and generated an initial set of codes. Four authors (KF, KM, LG, HA) reviewed and discussed the initial coding scheme; modifications and clarifying distinctions were made based on that discussion. Two authors (LG, HA) then independently coded all responses in Excel. These two authors met to compare codes and to identify disagreements and questions. Questions and disagreements which were not readily resolved were brought to the senior authors (KF and/or KM, both experienced qualitative researchers) for further discussion and resolution. We summarized the themes identified in reasons given for not seeking and receiving care, and themes in responses describing how care fell short for those who received care but rated the care as less than excellent. Coding of responses to open-ended items was performed in Excel.

## Results

### Response rate and respondent characteristics

There were 2,840 completed surveys among the 5,097 KnowledgePanel® members invited to respond, for an overall completion rate of 55.7%. The analytic sample for this study consists of 2,828 respondents who either responded to the question assessing long COVID awareness (*n* = 2,827) and/or responded affirmatively to experiencing symptoms consistent with long COVID (*n* = 272). Of these, the mean age was 50.4 years (range 18–94), approximately half were female (*n* = 1,493; 52.8%), 40.2% identified as Hispanic, 29.8% as Black, and 26.7% as White. Nearly one-fifth (*n* = 508; 18.0%) completed the survey in Spanish. Additional demographic characteristics are provided in Table 1.

**Table 1:** Respondent characteristics, according to long COVID awareness.

**Characteristic**		**Heard of long COVID**		***p*-value**
	**Overall sample** (*n* = 2,828)	**Yes** (*n* = 1,768)	**No / Not sure** (*n* = 1,059)	
Mean age, years, range (SD)*	50.4, 18–94 (17.1)	50.8, 18–94 (16.9)	49.7, 18–92 (17.5)	0.108
Age				0.089
18–29	409 (14.5)	244 (13.8)	165 (15.6)	
30–44	713 (25.2)	448 (25.3)	265 (25.0)	
45–59	730 (25.8)	439 (24.8)	291 (27.5)	
60+	976 (34.5)	637 (36.0)	338 (31.9)	
Gender				0.210
Male	1,335 (47.2)	851 (48.1)	484 (45.7)	
Female	1,493 (52.8)	917 (51.9)	575 (54.3)	
Education				<0.001
Less than high school	340 (12.0)	143 (8.1)	197 (18.6)	
High school diploma or GED	855 (30.2)	445 (25.2)	409 (38.6)	
Some college or Associate’s degree	756 (26.7)	490 (27.7)	266 (25.1)	
Bachelor’s degree	508 (18.0)	391 (22.1)	117 (11.1)	
Master’s degree or higher	369 (13.1)	299 (16.9)	70 (6.6)	
Race / ethnicity / survey completion language				<0.001
White, non-Hispanic	756 (26.7)	566 (32.0)	190 (17.9)	
Black, non-Hispanic	844 (29.8)	529 (29.9)	314 (29.7)	
Hispanic, completed survey in English	627 (22.2)	397 (22.5)	230 (21.7)	
Hispanic, completed survey in Spanish	508 (18.0)	203 (11.5)	305 (28.8)	
Other / More than 1 race, non-Hispanic	93 (3.3)	73 (4.1)	20 (1.9)	
Region				<0.001
Northeast	428 (15.1)	301 (17.0)	127 (12.0)	
Midwest	421 (14.9)	273 (15.4)	148 (14.0)	
South	1,277 (45.2)	760 (43.0)	516 (48.7)	
West	702 (24.8)	434 (24.6)	268 (25.3)	
Metropolitan statistical area (MSA) status				0.108
Metro	2,579 (91.2)	1,624 (91.9)	954 (90.1)	
Non-Metro	249 (8.8)	144 (8.1)	105 (9.9)	
Employment status				0.002
Working full-time	1,303 (46.1)	860 (48.6)	443 (41.8)	
Working part-time	379 (13.4)	231 (13.1)	148 (14.0)	
Not working	1,146 (40.5)	677 (38.3)	468 (44.2)	
Insurance status^†^				<0.001
No insurance	255 (9.3)	96 (5.6)	159 (15.8)	
Any insurance	2,481 (90.7)	1,635 (94.5)	846 (84.2)	
Household income				<0.001
Less than $10,000	165 (5.8)	62 (3.5)	103 (9.7)	
$10,000 to $49,999	871 (30.8)	434 (24.6)	437 (41.3)	
$50,000 to $74,999	489 (17.3)	290 (16.4)	199 (18.8)	
$75,000 to $149,999	796 (28.2)	575 (32.5)	221 (20.9)	
$150,000 or more	507 (17.9)	407 (23.0)	99 (9.4)	
History of COVID-19 infection^‡^				<0.001
Yes, had it once or more than once	1,365 (48.5)	909 (51.7)	455 (43.1)	
No, have not had COVID-19	1,220 (43.3)	721 (41.0)	499 (47.3)	
Not sure	232 (8.2)	130 (7.4)	102 (9.7)	
COVID-19 vaccination status^§^				<0.001
Received at least 1 dose	2,338 (82.9)	1,514 (85.9)	823 (78.0)	
Haven’t received any doses	481 (17.1)	249 (14.1)	232 (22.0)	

* *T*-tests used to compare mean age.

^†^ Missing insurance status, *n* = 91.

^‡^ Missing COVID-19 infection history, *n* = 11.

^§^ Missing vaccination status, *n* = 9.

### Long COVID awareness

Overall, 62.5% of respondents had heard of long COVID. Characteristics associated with having heard of long COVID on bivariate analysis are shown in Table 1. In multivariate analysis (Table 2), several factors were associated with increased odds of having heard of long COVID. For example, the odds of having heard of long COVID were more than twice as high in respondents with a master’s degree or higher (OR 2.21; 95% CI 1.49, 3.26) than in those without a high school diploma or GED, and those with an annual income of greater than $75,000 had a two-fold higher odds of having heard of long COVID (OR 2.08; 95% CI 1.38, 3.12) than those earning less than $10,000 annually. Additional factors associated with having heard of long COVID on multivariate analysis included having health insurance (OR 1.55; 95% CI 1.15, 2.08), a history of prior COVID-19 infection (OR 1.37; 95% C.I. 1.15, 1.64), and receipt of at least one dose of a COVID-19 vaccine (OR 1.34; 95% C.I. 1.06, 1.69). After multivariate adjustment, members of racial and ethnic minority groups remained significantly less likely to have heard of long COVID. Compared to White respondents, odds of having heard of long COVID were more than 25% lower among respondents who identify as Black (OR 0.64; 95% CI 0.51, 0.81), and odds of having heard of long COVID were 40% lower among those who identify as Hispanic and completed the survey in English (OR 0.59; 95% CI 0.46, 0.76). Respondents who identify as Hispanic and completed the survey in Spanish were least likely to have heard of long COVID (OR 0.31, 95% CI 0.23, 0.41) compared to White respondents who completed the survey in English.

**Table 2:** Associations between respondent characteristics and long COVID awareness, results from multivariable analysis.

**Characteristic**	**Adjusted odds ratio** (95% Confidence interval)	***p* value**
Age	1.00 (0.99, 1.00)	0.568
Gender		0.731
Male	Ref	
Female	1.03 (0.87, 1.22)	
Education		<0.001
No high school diploma or GED	Ref	
High school diploma or GED	1.07 (0.81, 1.41)	
Some college or Associate’s degree	1.43 (1.06, 1.93)	
Bachelor’s degree	2.19 (1.56, 3.07)	
Master’s degree or higher	2.21 (1.49, 3.26)	
Race/ethnicity/language		<0.001
White, non-Hispanic	Ref	
Black, non-Hispanic	0.64 (0.51, 0.81)	
Hispanic, completed survey in English	0.59 (0.46, 0.76)	
Hispanic, completed survey in Spanish	0.31 (0.23, 0.41)	
Other, non-Hispanic / 2+ races, non-Hispanic	1.02 (0.58, 1.80)	
Metropolitan statistical area status		0.081
Metro	Ref	
Non-metro	0.77 (0.57, 1.03)	
Insurance status		0.004
No insurance	Ref	
Any type of insurance	1.55 (1.15, 2.08)	
Household income		<0.001
Less than $10,000	Ref	
$10,000 to $49,999	1.25 (0.86, 1.84)	
$50,000 to $74,999	1.44 (0.96, 2.17)	
$75,000 to $149,999	2.08 (1.38, 3.12)	
$150,000 or more	2.34 (1.49, 3.70)	
History of COVID-19 infection		0.002
Have not had COVID	Ref	
Yes, I had it once or more than once	1.37 (1.15, 1.64)	
Not sure	1.06 (0.76, 1.47)	
COVID-19 vaccination status		0.014
Have not received any vaccine doses	Ref	
Received at least 1 vaccine dose	1.34 (1.06, 1.69)	

### Receipt and experience of medical care for long COVID

Of the 1,365 respondents who reported a history of COVID-19 infection, 272 (19.9%) reported symptoms consistent with long COVID (any symptom lasting longer than 1 month). Of those with symptoms consistent with long COVID, 25.4% (*n* = 68) had not heard of it. Nearly half (*n* = 132; 48.9%) reported a symptom duration of 1–3 months, and most were either not limited at all (*n* = 88; 32.5%) or limited a little (*n* = 150; 55.4%) by their long COVID symptoms (Table 3). Approximately one-quarter (*n* = 72; 26.8%) reported having been seen by a healthcare provider for their symptoms of long COVID. The most common reasons for not having been seen by a healthcare provider for symptoms of long COVID included that it did not occur to the respondent to seek care (*n* = 121), their symptoms were mild (*n* = 15), they expected their symptoms would get better with time (*n* = 15), they did not think anything could be done (*n* = 7), they could not afford an appointment (*n* = 7), there was a long wait for an appointment (*n* = 5), and they were too busy with competing health issues or had not prioritized seeking care (*n* = 5).

**Table 3:** Receipt of healthcare among respondents with symptoms consistent with long COVID.

		**Receipt of medical care for long COVID symptoms**		
Characteristic	Overall *N* = 272	Seen by provider *N* = 72	Have not sought care or not seen by provider *N* = 197	*P*-value
Age, mean, range (SD)	48.4, 20–82, (14.2)	52.7, 26–80 (14.0)	47.0, 20–82 (13.9)	0.003
Age, years				0.022
18–29	28 (10.3)	3 (4.2)	24 (12.2)	
30–44	86 (31.6)	23 (31.9)	62 (31.5)	
45–59	94 (34.6)	21 (29.2)	73 (37.1)	
60+	64 (23.5)	25 (34.7)	38 (19.3)	
Gender				0.563
Male	101 (37.1)	25 (34.7)	76 (38.6)	
Female	171 (62.9)	47 (65.3)	121 (61.4)	
Education				0.106
Less than high school	35 (12.9)	9 (12.5)	25 (12.7)	
High school diploma or GED	78 (28.7)	15 (20.8)	61 (31.0)	
Some college or Associate’s degree	83 (30.5)	30 (41.7)	53 (26.9)	
Bachelor’s degree	48 (17.7)	9 (12.5)	39 (19.8)	
Master’s degree or higher	28 (10.3)	9 (12.5)	19 (9.6)	
Race / ethnicity / language				0.070
White, non-Hispanic	69 (25.4)	23 (31.9)	46 (23.4)	
Black, non-Hispanic	52 (19.1)	19 (26.4)	31 (15.7)	
Hispanic, completed survey in English	85 (31.3)	17 (23.6)	68 (34.5)	
Hispanic, completed survey in Spanish	57 (21.0)	12 (16.7)	44 (22.3)	
Other / More than 1 race, non-Hispanic	9 (3.3)	1 (1.4)	8 (4.1)	
Region				0.769
Northeast	45 (16.5)	14 (19.4)	30 (15.2)	
Midwest	40 (14.7)	10 (13.9)	30 (15.2)	
South	113 (41.5)	31 (43.1)	81 (41.1)	
West	74 (27.2)	17 (23.6)	56 (28.4)	
Metropolitan statistical area (MSA) status				0.063
Metro	250 (91.9)	70 (97.2)	178 (90.4)	
Non-metro	22 (8.1)	2 (2.8)	19 (9.6)	
Employment status				0.720
Working full-time	144 (52.9)	37 (51.4)	107 (54.3)	
Working part-time	34 (12.5)	8 (11.1)	26 (13.2)	
Not working	94 (34.6)	27 (37.5)	64 (32.5)	
Insurance status*				0.034
No insurance	34 (12.7)	4 (5.6)	30 (15.5)	
Any insurance	234 (87.3)	67 (94.4)	164 (84.5)	
Household income				0.666
Less than $10,000	18 (6.6)	3 (4.2)	14 (7.1)	
$10,000 to $49,999	90 (33.1)	20 (27.8)	68 (34.5)	
$50,000 to $74,999	51 (18.8)	15 (20.8)	36 (18.3)	
$75,000 to $149,999	69 (25.4)	20 (27.8)	49 (24.9)	
$150,000 or more	44 (16.2)	14 (19.4)	30 (15.2)	
Have you heard of long COVID				0.584
Yes	200 (74.6)	52 (72.2)	148 (75.5)	
No or not Sure	68 (25.4)	20 (27.8)	48 (24.5)	
COVID-19 vaccination status^†^				0.038
Received at least 1 dose	219 (80.8)	64 (88.9)	152 (77.6)	
Haven’t received any doses	52 (19.2)	8 (11.1)	44 (22.5)	
Long COVID symptom duration^‡^				0.017
1-3 months	132 (48.9)	29 (40.3)	102 (52.3)	
3–6 months	55 (20.4)	14 (19.4)	40 (20.5)	
6–12 months	30 (11.1)	6 (8.3)	24 (12.3)	
More than 12 months	53 (19.6)	23 (31.9)	29 (14.9)	
Long COVID symptom impact^†^				0.010
Not limited at all	88 (32.5)	15 (21.1)	73 (37.1)	
Limited a little	150 (55.4)	42 (59.2)	106 (53.8)	
Limited a lot	33 (12.2)	14 (19.7)	18 (9.1)	

* Missing insurance status, long COVID awareness, *n* = 4.

^†^ Missing vaccination status, long COVID symptom impact, *n* = 1.

^‡^ Missing long COVID symptom duration, *n* = 2.

Factors associated with having seen a healthcare provider for symptoms of long COVID on bivariate analysis are shown in Table 3. In multivariate analysis, factors associated with having been seen by a healthcare provider for long COVID included older age (OR 1.03; 95% CI 1.01, 1.05), symptom duration longer than 12 months (OR 2.62; 95% CI 1.24, 5.57), and being limited “a lot” by long COVID symptoms (OR 3.44; 95% CI 1.28, 9.28) (Table 4).

**Table 4:** Associations between respondent characteristics and receipt of medical care for long COVID symptoms, results from multivariable analysis.

**Characteristic**	**Adjusted odds ratio** (95% Confidence interval)	***P* value**
Age	1.03 (1.01, 1.05)	0.013
Gender		0.331
Male	Ref	
Female	1.37 (0.73, 2.59)	
Race/ethnicity/language		0.408
White, non-Hispanic	Ref	
Black, non-Hispanic	1.32 (0.57, 3.05)	
Hispanic, completed survey in English	0.64 (0.28, 1.44)	
Hispanic, completed survey in Spanish	0.66 (0.27, 1.62)	
Other, non-Hispanic / 2+ races, non-Hispanic	0.46 (0.05, 4.16)	
Metropolitan statistical area status		0.084
Metro	Ref	
Non-metro	0.25 (0.05, 1.20)	
Insurance status		0.200
No insurance	Ref	
Any insurance	2.16 (0.67, 7.00)	
Long COVID symptom duration		0.065
1–3 months	Ref	
3–6 months	1.41 (0.64, 3.10)	
6–12 months	0.87 (0.30, 2.50)	
More than 12 months	2.62 (1.24, 5.57)	
Long COVID symptom impact		0.050
Not limited at all	Ref	
Limited a little	1.65 (0.81, 3.36)	
Limited a lot	3.44 (1.28, 9.28)	

Of the respondents with symptoms consistent with long COVID who had been seen by a healthcare provider for these symptoms (*n* = 72), approximately one-half (47.2%) reported being diagnosed with long COVID, and 14 (19.4%) were referred for additional medical care elsewhere. Most (56/72; 77.8%) rated the care they received as less than excellent. Of those who provided a reason why care fell short (*n* = 30), the most common reason (*n* = 19) was dissatisfaction with the lack of knowledge (“Her answer was ‘not a lot is known about long COVID.’ What do you do with that answer?”) and/or treatments (“I feel like I still have symptoms and there are no medications to heal it”) for long COVID. Other reasons included feeling like the provider did not listen, take them seriously, or was dismissive (“the cough is still there to this day and the physician ignored or never addressed my concerns”; *n* = 7), and wanting more testing or evaluation of their symptoms (*n* = 4). Most respondents (*n* = 61; 84.7%) somewhat or strongly agreed that the provider they saw for symptoms of long COVID took them seriously, but some (*n* = 11; 15.3%) disagreed.

## Discussion

In this large survey that oversampled Black and Hispanic respondents, we found low rates of awareness of long COVID; nearly four in ten respondents had not heard of long COVID. Although there are no benchmarks for acceptable levels of public awareness, the limited awareness in this survey is striking given the magnitude of the COVID-19 pandemic overall and of long COVID specifically. As many as 35% of people with COVID-19 infection report having symptoms more than 60 days after infection (26), and the economic burden of long COVID is estimated to be a staggering $3.7 trillion (27), making it a major public health challenge. In the absence of currently approved treatments specific for long COVID, public awareness is critical to encouraging preventative behaviors, such as vaccination and treatment with oral antivirals which both appear to reduce the risk of developing long COVID or post-COVID conditions (PCC) (28–30). Indeed, in the present study, we found that long COVID awareness is positively associated with COVID-19 vaccination status supporting the idea that awareness of long COVID may be an important lever for motivating and promoting uptake of preventative behaviors.

In addition to low overall awareness, we also found marked disparities in awareness of long COVID according to race, ethnicity, and language, even after controlling for other social determinants of health such as education and income. Long COVID awareness was lowest among Hispanic respondents who completed the survey in Spanish. Although the present study was only conducted in English and Spanish, our findings suggest that long COVID awareness may also be low among other populations with limited English proficiency in the US. Low long COVID awareness in these populations may compound existing disparities in receipt of COVID-19 boosters (31–33) and oral antivirals (19). Concerted efforts to increase awareness of long COVID tailored for communities with limited English proficiency and members of racial and ethnic minority groups are needed.

Although long COVID awareness may be important for promoting preventative behaviors, it does not appear to drive healthcare utilization for long COVID. We did not find an association between long COVID awareness and receipt of medical care for long COVID symptoms. Our quantitative and qualitative results indicate that receipt of medical care for long COVID is primarily driven by both the duration and impact of symptoms suggesting that patients with protracted and/or severe symptoms seek care regardless of whether they are aware that their symptoms could be consistent with long COVID. At the same time, a quarter of patients with symptoms consistent with long COVID had not heard of it. Even if their symptoms may be less severe, medical care can still offer a diagnosis which for some patients can provide validation of their experience of symptoms (34), an additional potential benefit of increased public awareness.

Among the subset of respondents with symptoms consistent with long COVID who received care for these symptoms, only 22.2% rated their care for long COVID as excellent, indicating a need to improve the clinical care of patients with long COVID. A common reason for rating care as less than excellent was the perception that the provider had inadequate knowledge about long COVID. We also found a substantial minority of respondents who reported the provider was dismissive of their symptoms, consistent with other reports of patients with long COVID symptoms describing “medical gaslighting” (35). The proportion of respondents in the present study describing dismissive providers (15.8%) is lower than in a prior study (34%) conducted in 2021 (35). This difference may reflect improved provider knowledge and understanding about long COVID more recently or may simply be due to differences in populations sampled. The experiences of medical care by respondents with symptoms of long COVID in our study highlight the need for increased primary care provider education about long COVID, as has been called for in other reports (36–39).

The primary strength of this study is the large and diverse sample that allowed us to assess multiple aspects of long COVID from awareness to receipt of and experience of medical care for long COVID symptoms, including among non-primary English speakers. This study also has limitations. Because we oversampled specific populations to achieve diversity, the estimate of long COVID awareness is not nationally representative and does not include all racial groups. We relied on respondent’s self-report of history of COVID-19 infection which could result in misclassification. However, this is consistent with how patients with potential long COVID will come to medical attention. Although we used the CDC’s definition of long COVID, a universal challenge related to studying long COVID is the multiple definitions that rely on symptom report. Because our goal was not to describe the prevalence of long COVID, this limitation is not central to our findings. Further, our finding that 19.9% of respondents with a history of COVID-19 infection reported symptoms consistent with long COVID is in keeping with estimates from other US studies that have found symptoms lasting longer than 4 weeks among 10–30% of people with COVID-19 (36). Despite the large sample size, only a small proportion of respondents received care for long COVID symptoms limiting the conclusions we can draw about receipt of and experience of medical care for long COVID.

## Conclusion

This large survey with oversampling of populations at risk of disparities in the US, documents limited overall awareness of long COVID and marked disparities in awareness among members of racial/ethnic minority groups and those with limited English proficiency. Reducing the risk of long COVID may be an important motivator for promoting uptake of preventative behaviors. Low public awareness of long COVID could reduce the impact of this as a lever for mitigating the major public health burden of long COVID. Public health efforts focused on building awareness of long COVID among members of racial/ethnic minority groups and those with limited English proficiency are needed.

## Data availability statement

The datasets in this article will be made available upon reasonable request to the corresponding author and with an executed data use agreement in place. Requests to access the datasets should be directed to Kimberly.Fisher2@umassmed.edu.

## Ethics statement

The studies involving humans were approved by the Boston University Medical Center Institutional Review Board. The studies were conducted in accordance with the local legislation and institutional requirements. The ethics committee/institutional review board waived the requirement of written informed consent for participation from the participants or the participants’ legal guardians/ next of kin because this study was deemed exempt by the reviewing IRB. As such, we provided participants with abbreviated consent language before they began study activities.

## Author contributions

KF: Conceptualization, Data curation, Formal analysis, Investigation, Methodology, Project administration, Supervision, Validation, Writing – original draft. KM: Conceptualization, Data curation, Formal analysis, Investigation, Methodology, Supervision, Writing – original draft. ME: Conceptualization, Methodology, Writing – original draft. LG: Data curation, Investigation, Writing – review & editing. HA: Data curation, Investigation, Writing – review & editing. YZ: Formal analysis, Writing – review & editing. SC: Formal analysis, Writing – review & editing. JM: Writing – review & editing, Conceptualization. BL: Conceptualization, Funding acquisition, Writing – review & editing.
