# Supplementary material for: Long COVID awareness and receipt of medical care: a survey among populations at risk for disparities
Source: Front Public Health. 2024 May 30;12:1360341. doi: 10.3389/fpubh.2024.1360341 (PMC11173587; doi:10.3389/fpubh.2024.1360341)
Supplement: Supplementary file 1 [file Table_1.DOCX]

**Supplementary Table 1.** Long COVID survey items

| **Question** | **Response options** |
| --- | --- |
| The following items were asked of all respondents. | |
| **Vaccination history** |  |
| Have you received at least one dose of the COVID-19 vaccine? | - Yes, got a one-dose vaccine (J&J) - Yes, got the first dose of a two-dose vaccine (Moderna or Pfizer) - Yes, got both doses of a two-dose vaccine (Moderna or Pfizer) - No, have not gotten the vaccine |
| ***If “Yes, got one-dose vaccine (J&J)” or “Yes, got both doses of two-dose vaccine (Moderna or Pfizer****)”:*  Have you gotten the updated COVID-19 booster? This is sometimes referred to as a “bivalent” vaccine. If you got boosted after September 1, 2022, this is probably the vaccine you got.* | - Yes - No - Not sure |
| ***If “No”, haven’t gotten the updated COVID-19 booster:***  Do you intend to get the updated COVID-19 booster?* | - Yes - No - Not sure |
| ***If “Yes”, intend to get the updated booster:***  Tell us why you haven’t gotten the updated booster yet?* | Open-ended. |
| ***If “Not sure” whether intend to get the updated booster:***  Tell us why you are not sure whether you will get an updated booster.* | Open ended. |
| ***If “No”, don’t intend to get the updated booster:***  Tell us why you don’t intend to get an updated booster.* | Open-ended. |
| **Long COVID awareness** |  |
| Have you heard of **long COVID**?  *Also referred to as post-COVID, Long-haul COVID, Post-acute COVID-19, or Chronic COVID.* | - Yes - No - Not sure |
| **Definition of long COVID** *(provided to all participants after responding to prior question):*  Long COVID refers to health problems people can experience four or more weeks after having COVID-19. These can be new health problems or worsening of health problems that people had before getting COVID-19. Even people who didn’t have symptoms of COVID-19 can have long COVID. There are lots of different types of symptoms of long COVID. They can last for different lengths of time. | |
| **Please indicate how much you agree or disagree with the following statements:**  There’s not really anything you can do to reduce your chances of getting long COVID.*  Long COVID can be serious and interfere with day-to-day activities.*  Long COVID is a good reason to keep up to date on COVID-19 vaccinations.* | - Strongly agree - Agree - Disagree - Strongly disagree |
| How much do you trust the healthcare system to do what’s right for you? | - Do not trust at all - Trust a little - Trust somewhat - Trust a great deal |
| **COVID-19 history and symptoms** | |
| Have you had COVID-19? **Select one.** | - Yes, I had it once - Yes, I had it more than once - No, I have not had COVID-19 - Not sure |
| The following items were asked of respondents who reported a history of COVID-19 infection only. | |
| ***If “Yes, I had it once” or “Yes, I had it more than once”:***  **When** did you last have COVID-19? | - Less than 1 month ago - 1-3 months ago - 3-6 months ago - 6-12 months ago - More than 12 months ago |
| ***If “Yes, I had it once” or “Yes, I had it more than once”:***  For **any** time you had COVID-19, have you experienced any COVID-19 symptoms that have lasted longer than a month? | - Yes - No - Not sure - I didn’t have any symptoms when I had COVID |
| The following items were asked of respondents who reported symptoms consistent with long COVID (symptoms lasting longer than one month) only. | |
| ***If “Yes”, symptoms lasted more than one month:***  Which symptoms did you experience for longer than one month? **Select all that apply.** | - Cough - Feeling tired / Fatigue - Stuffy nose / Congestion - Trouble breathing / Shortness of breath - Loss of taste or smell - Body aches - Trouble concentrating - Trouble sleeping - Mood changes or anxiety - Stomach pain - Headaches - Heart racing / Heart palpitations - Joint or muscle pain - Dizziness - Other: _______________________ |
| ***If “Yes”, symptoms lasted longer than one month:***  How long did your symptoms last? | - 1-3 months - 3-6 months - 6-12 months - More than 12 months |
| ***If “Yes”, symptoms lasted longer than one month:***  Are you still having any symptoms of long COVID? | - Yes - No - Not sure |
| ***If “Yes”, symptoms lasted longer than one month*** *(branching based on whether still present or not):*  [Did/Do] your long COVID symptoms limit your ability to do the things you usually do? | - No, not limited at all - Yes, limit[ed] a little - Yes, limit[ed] a lot |
| **Medical care seeking/barriers** |  |
| ***If “Yes”, symptoms lasted longer than one month:***  Have you tried to get medical care for any of the symptoms that lasted more than a month? | - No, it didn’t occur to me to get care for long COVID symptoms - No, I thought about getting care but I didn’t pursue it. - Yes, I tried to get care for my long COVID symptoms but never saw a provider - Yes, I have an appointment for my long COVID symptoms but have not yet been seen.   Yes, I have been seen by a provider for my long COVID symptoms |
| ***If “No, I thought about getting care but didn’t pursue it”:***  Why didn’t you pursue care for your long covid symptoms? | Open-ended. |
| ***If “Yes, I tried to get care for my long COVID symptoms but never saw a provider”:***  Why weren’t you able to see a provider for your long COVID symptoms? | Open-ended. |
| ***If “Yes, I have been seen by a provider for my long COVID symptoms”:***  **Please indicate how much you agree with the following statement:**  The healthcare provider I saw took my symptoms seriously. | - Strongly agree - Somewhat agree - Somewhat disagree - Strongly disagree |
| ***If “Yes, I have been seen by a provider for my long COVID symptoms”***  The healthcare provider I saw told me I might have long COVID (or *post-COVID, Long-haul COVID, Post-acute COVID-19, or Chronic COVID)* | - Yes - No |
| ***If “Yes, I have been seen by a provider for my long COVID symptoms”***  The healthcare provider I saw referred me somewhere else for more medical care. | - Yes - No |
| ***If “Yes, I have been seen by a provider for my long COVID symptoms”***  Overall, how would you rate the medical care you received for your long COVID symptoms? | - Excellent - Very good - Good - Fair - Poor |
| ***If rated care as “Very good”, “Good”, “Fair”, “Poor”, or refused the previous question:***  Please tell us how your care fell short, and what should be done differently in the future: | Open-ended. |
| **Non-traditional care** |  |
| ***If “Yes”, symptoms lasted longer than one month:***  What, if anything, have you done to try to treat your long COVID symptoms (other than seeking medical care)?* | Open-ended. |
| **Social experience** |  |
| ***If “Yes”, symptoms lasted longer than one month:***  How have your friends and family reacted to your having long COVID symptoms?* | Open-ended. |
| ***If “Yes”, symptoms lasted longer than one month:***  How have your employer and/or co-workers reacted to your having long COVID symptoms?* | Open-ended. |
| The following item was asked of all respondents. | |
| **Health insurance** |  |
| What kind of health insurance or health care plan do you have, if any? (Select all that apply.) | - I don’t have health insurance - Private health insurance through a job or school - Insurance bought through a government exchange such as healthcare.gov - Insurance bought from a health plan or company - Medicare - Medi-Gap - Medicaid - CHIP or kid’s state insurance - Military health care - Indian Health Service - Other: ___________ - Don’t Know |

***** These items were examined and determined not to be relevant or informative to the current analyses and so are not reported in this manuscript.
